# Supplementary material for: Caldera resurgence driven by magma viscosity contrasts
Source: Nat Commun. 2017 Nov 24;8:1750. doi: 10.1038/s41467-017-01632-y (PMC5701002; doi:10.1038/s41467-017-01632-y)
Supplement: Supplementary file 1 — Supplementary Information [file 41467_2017_1632_MOESM1_ESM.pdf]

Caldera	CO_2 (ton yr^{-1})x10^4
**Yellowstone (U.S.A.)[2]**	985.50
**Campi Flegrei (Italy)[3]**	54.75
**Ischia (Italy)[4]**	45
Rotorua (New Zeland)[5]	36.5
**Pantelleria (Italy)[6]**	32
**Siwi (Tanna Island)[7]**	30.66
Fournas (Portugal, Azzorre)[8]	26.79
**Sierra Negra (Ecuador, Galapagos)[9]**	22.08
**Iwojima (Japan)[10]**	16.43
Latera (Italy)[11]	12.78
Pululahua (Ecuador)*[12]	9.86
Miyakeijma, (Japan)[13]	4.73
Usu (Japan)[14]	4.38
Cuicocha (Ecuador)*[12]	3.87
Nisyros (Greece)*[15]	3.07
Hakkoda (Japan)*[16]	2.70
Satsuma–Iwojima (Japan)[17]	0.73

Supplementary Table 1 – *Magmatic contribution of CO_2 soil degassing at several semi-plugged (or degassing*[1]*) calderas (resurgent calderas in bold); * = no distinction between magmatic and biogenic contribution of CO_2. In all the cited studies the magmatic component of the diffused CO_2 has been related to the crystallization of new magma input.*

Model	*p* (m)	*D* (m)	*d* (m)	η_{sil} (Pa s)	ρ_s (kg m^{-3})	*T* (°C)	$\rho_{o.v.}$ (kg m^{-3})	$\eta_{o.v.}$ (Pa s)	*v* (m s^{-1})	*t* (s)
**RIS 0**	0.04	-	-	-	1400	50	907.8	1.87×10^{-2}	1.5	290
**RIS 1**	0.03	-	-	-	1400	50	907.8	1.87×10^{-2}	1.5	60
**RIS 2**	0.03	0.17	0.015	10^4	1400	50	907.8	1.87×10^{-2}	1.19	180
**RIS 4**	0.03	0.12	0.015	10^4	1400	50	907.8	1.87×10^{-2}	1.19	225
**RIS 5**	0.03	0.06	0.015	10^4	1400	50	907.8	1.87×10^{-2}	1.19	50
**RIS 7**	0.03	0.06	0.015	10^4	1400	36.4	936.8	3.08×10^{-2}	1.19	65
**RIS 9**	0.07	0.08	0.06	10^4	1400	38	931	2.90x10^{-2}	0.88	248

Supplementary Table 2 *– Analogue experiments and related tested parameters. P = depth of the nozzle; D = diameter of silicone layer; d = depth of silicone level; η_{sil} = viscosity of silicone; ρ_s = density of silicone; T = temperature of vegetable oil; $\rho_{o.v.}$= density of vegetable oil; $\eta_{o.v.}$ = viscosity of vegetable oil; v = injection velocity of vegetable oil; t = duration of experiment.*

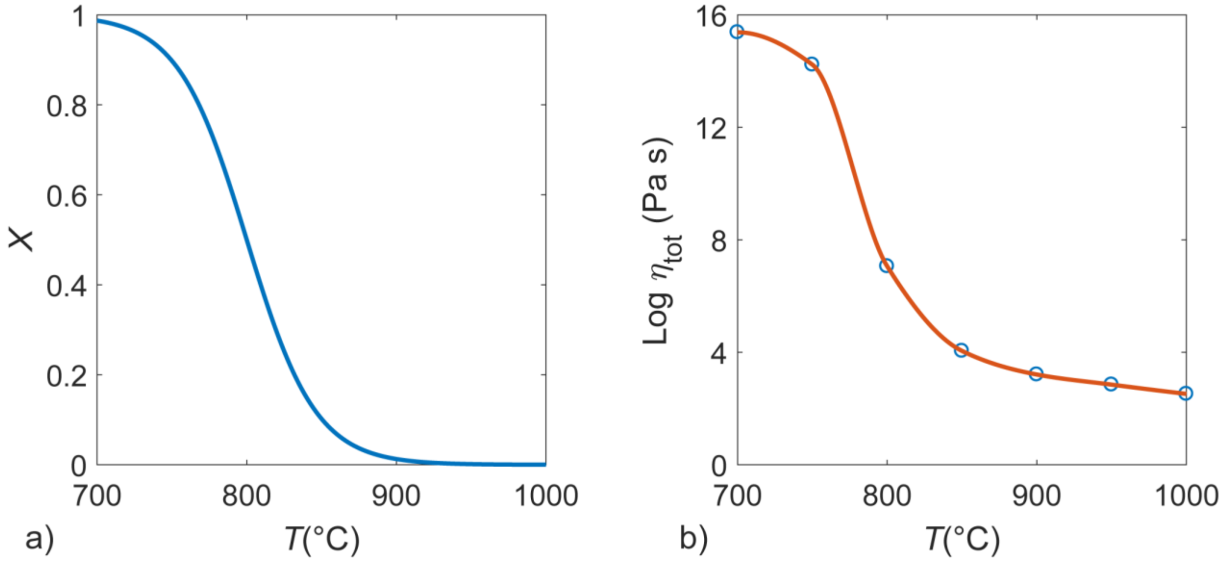

Supplementary Figure 1 *– Thermal models. a) Crystal fraction (X) as a function of temperature (T). b) Variation of the total viscosity (η_{tot}) with temperature (T).*

T (°C)	X	Log η_m (GRD) (Pa s)	Log η_r (Pa s)	Log η_{tot} (Pa s)
1000	0	2.52	0	2.52
950	0	2.85	0	2.85
900	0.01	3.20	0.01	3.21
850	0.10	3.92	0.13	4.05
800	0.50	4.71	2.35	7.06
750	0.90	5.43	8.80	14.23
700	0.99	5.96	9.42	15.38

Supplementary Table 3 - *Thermal models. T = temperature of the melt (in °C); X = crystal fraction of the magma; η_m = viscosity of the melt obtained with the GRD model[31]; η_r = relative viscosity obtained with a previous model[29]; η_{tot} = total viscosity.*

	Experiments	Nature
$\Pi_1 = (\rho_s\, gD)/c$	7.8-2.7	13- 4
$\Pi_2 = d/D$	$(1.3\text{-}7.5)\times10^{-1}$	$(1.9\text{-}3.8)\times10^{-1}$
$\Pi_3 = d/p$	$(5\text{-}8.6)\times10^{-1}$	5×10^{-1}
$\Pi_4 = p/D$	$(2.5\text{-}8.8)\times10^{-1}$	$(3.8\text{-}8.8)\times10^{-1}$
$\Pi_5 = \rho_{o.v.}/\ \rho_s$	$(6.3\text{-}6.7)\times10^{-1}$	8.5×10^{-1}
$\Pi_6 = \eta_{o.v.}/\ \eta_{sil}$	$(1.87\text{-}3.08)\times10^{-6}$	$(1\text{-}3)\times10^{-6}$
$\Pi_7 = (vt)/D$	$(1.3\text{-}3.5)\times10^{3}$	$(5.6\text{-}9.2)\times10^{3}$
$\Pi_8 = (\rho_{o.v.}\, p^2)/(\ \eta_{o.v}\, t)$	$(1.9\text{-}6.7)\times10^{-1}$	$(1.5\text{-}4.5)\times10^{-3}$

***Supplementary Table 4** – Analogue experiments. The eight dimensionless parameters in experiments and nature*

Supplementary References

1. Acocella, V., Di Lorenzo, R., Newhall, C. & Scandone, R. An overview of recent (1988 to 2014) caldera unrest: Knowledge and perspectives. *Rev. Geophys.* **53**, doi:10.1002/ 2015RG000492. (2015)
2. Werner, C. & Brantley, S. CO_2 Emissions from the Yellowstone volcanic system. *Geochem. Geophys. Geosyst.* **4**, 1061 doi:10.1029/2002GC000473 (2003)
3. Chiodini, G. *et al.* Evidence of thermal-driven processes triggering the 2005–2014 unrest at Campi Flegrei caldera. *Earth Planet. Sci. Lett.* **414**, 58-67 (2015)
4. Di Napoli, R. *et al.* A model for Ischia hydrothermal system: evidences from the chemistry of thermal groundwaters. *J. Volcanol. Geotherm. Res.* **186**, 133-159 (2009).
5. Werner, C. & Cardellini, C. Comparison of carbon dioxide emissions with fluid upflow, chemistry, and geologic structures at the Rotorua geothermal system, New Zealand. *Geothermics* **35**, 221-238 (2006).
6. Favara, R., Giammanco, S., Inguaggiato, S. & Pecoraino, G. Preliminary estimate of CO_2 Output from Pantelleria Island volcano (Sicily, Italy): evidence of active mantle degassing. *Appl. Geochem.* **16**, 883 – 894 (2001)
7. Métrich, N. et al. Magma and volatile supply to post-collapse volcanism and block resurgence in Siwi Caldera (Tanna Island, Vanuatu arc). *J. Petrol.* **52**, 1077-1105 (2011)
8. Viveiros, F. *et al.* CO_2 Emissions at Furnas volcano, São Miguel Island, Azores archipelago: Volcano monitoring perspectives, geomorphologic studies, and land use planning application. *J. Geophys. Res.* **115**, B12208, doi:10.1029/2010JB007555 (2010)
9. Padrón, E. *et al.* Fumarole/plume and diffuse CO_2 emission from Sierra Negra caldera, Galapagos archipelago. *Bull. Volcanol.* **74**, 1509–1519 (2012)
10. Notsu, K., *et al.* Diffuse CO_2 efflux from Iwojima volcano, Izu-Ogasawara arc, Japan. *J. Volcanol. Geotherm. Res.* **139**, 147–161 (2005)
11. Chiodini, G. *et al.* Carbon dioxide degassing at Latera caldera (Italy): evidence of geothermal reservoir and evaluation of its potential energy. *J. Geophys. Res.* **112**, B12204 doi:10.1029/2006JB004896 (2007)

12. Padrón, E. *et al.* Diffuse CO_2 emission rate from Pululahua and the lake-filled Cuicocha calderas, Ecuador. *J. Volcanol. Geotherm. Res.* **176**, 163-169 (2008)
13. Hernández, P.A., *et al.* Diffuse emission of CO_2 from Miyakejima volcano, Japan. *Chem. Geol.* **177**, 175-185 (2001)
14. Hernández, P.A. *et al.* Carbon dioxide degassing by advective flow from Usu volcano, Japan. *Science* **292**, 83-86 (2001)
15. Cardellini, C., Chiodini, G. & Frondini, F. Application of stochastic simulation to CO_2 flux from soil: mapping and quantification of gas release. *J. Geophys. Res.: Solid Earth* **108**, 2425 doi:10.1029/2002JB002165 (2003)
16. Hernandez, P.A. *et al.* Carbon dioxide emissions from soils at Hakkoda, north Japan. *J. Geophys. Res.* **108**, 2210, doi:10.1029/2002JB001847 (2003)
17. Shimoike, Y., Kazahaya, K. & Shinohara, H. Soil gas emission of volcanic CO_2 at Satsuma-Iwojima volcano, Japan. *Earth Planets Space* **54**, 239-247 (2002)
